# Associations of genetically proxied inhibition of HMG-CoA reductase, NPC1L1, and PCSK9 with breast cancer and prostate cancer

**DOI:** 10.1186/s13058-022-01508-0

**Published:** 2022-02-12

**Authors:** Lulu Sun, Huan Ding, Yiming Jia, Mengyao Shi, Daoxia Guo, Pinni Yang, Yu Wang, Fanghua Liu, Yonghong Zhang, Zhengbao Zhu

**Affiliations:** 1 Department of Epidemiology, School of Public Health and Jiangsu Key Laboratory of Preventive and Translational Medicine for Geriatric Diseases, Medical College of Soochow University, 199 Renai Road, Industrial Park District, Suzhou, 215123 Jiangsu Province China; 2 Department of Chronic Infectious Disease Control and Prevention, Wuxi Center for Disease Control and Prevention, Wuxi, China; 3 Department of Epidemiology, Tulane University School of Public Health and Tropical Medicine, New Orleans, LA USA; 4 School of Nursing, Medical College of Soochow University, Suzhou, China

**Keywords:** LDL-C-lowering drug, HMG-CoA reductase, NPC1L1, PCSK9, Breast cancer, Prostate cancer

## Abstract

**Background:**

Preclinical and epidemiological studies indicate a potential chemopreventive role of low-density lipoprotein cholesterol (LDL-C) -lowering drugs in the risks of breast cancer and prostate cancer, but the causality remains unclear. We aimed to evaluate the association of genetically proxied inhibition of 3-hydroxy-3-methylglutaryl coenzyme A (HMG-CoA) reductase, Niemann-Pick C1-Like 1 (NPC1L1), and proprotein convertase subtilisin/kexin type 9 (PCSK9) with risks of breast cancer and prostate cancer using a two-sample Mendelian randomization (MR) method.

**Methods:**

Single-nucleotide polymorphisms (SNPs) in *HMGCR*, *NPC1L1*, and *PCSK9* associated with LDL-C in a genome-wide association study (GWAS) meta-analysis from the Global Lipids Genetics Consortium (GLGC; up to 188,577 European individuals) were used to proxy inhibition of HMG-CoA reductase, NPC1L1, and PCSK9. Summary statistics with outcomes were obtained from a GWAS meta-analysis of the Breast Cancer Association Consortium (BCAC; 228,951 European females) and a Prostate Cancer Association Group to Investigate Cancer Associated Alterations in the Genome (PRACTICAL; 140,254 European males) consortium. SNPs were combined into multiallelic models and MR estimates representing lifelong inhibition of targets were generated using the inverse-variance weighted method.

**Results:**

Genetically proxied inhibition of HMG-CoA reductase (OR: 0.84; 95% CI 0.74–0.95; *P* = 0.005) and NPC1L1 (OR: 0.72; 95% CI 0.58–0.90; *P* = 0.005) equivalent to a 1-mmol/L (38.7 mg/dL) reduction in LDL-C was associated with reduced breast cancer risk. There were no significant associations of genetically proxied inhibition of PCSK9 with breast cancer. In contrast, genetically proxied inhibition of PCSK9 (OR: 0.81; 95% CI 0.73–0.90; *P* < 0.001) but not HMG-CoA reductase and NPC1L1 was negatively associated with prostate cancer. In the secondary analysis, genetically proxied inhibition of HMG-CoA reductase (OR: 0.82; 95% CI 0.71–0.95; *P* = 0.008) and NPC1L1 (OR: 0.66; 95% CI 0.50–0.86; *P* = 0.002) was associated with estrogen receptor-positive breast cancer, whereas there was no association of HMG-CoA reductase and NPC1L1 with estrogen receptor-negative breast cancer.

**Conclusions:**

Genetically proxied inhibition of HMG-CoA reductase and NPC1L1 was significantly associated with lower odds of breast cancer, while genetically proxied inhibition of PCSK9 was associated with reduced risk of prostate cancer. Further randomized controlled trials are needed to confirm the respective roles of these LDL-C-lowering drugs in breast cancer and prostate cancer.

**Supplementary Information:**

The online version contains supplementary material available at 10.1186/s13058-022-01508-0.

## Introduction

There were 19.3 million new cancer cases and almost 10.0 million cancer deaths worldwide in 2020, which caused an enormous economic burden for patients and society [1]. Breast cancer was the most common cancer among females, while prostate cancer was the second most frequently occurring cancer among males [1]. Although these two cancers arise in different organs in terms of anatomy and physiological function, they are typically hormone-dependent and have remarkable underlying biological similarities [2], because both organs require gonadal steroids for their development. With the limited treatment measures (e.g., surgery, radiation and hormone therapy) and poor prognosis of breast cancer and prostate cancer [2–4], primary prevention potentially offers the most cost-effective strategy for breast cancer and prostate cancer control and would effectively reduce the disease burden.

3-hydroxy-3-methylglutaryl coenzyme A (*HMG-CoA*; target of statins) reductase, Niemann-Pick C1-Like 1 *(NPC1L1*; target of ezetimibe) and proprotein convertase subtilisin/kexin type 9 (*PCSK9*; target of PCSK9 inhibitors) are common targets of low-density lipoprotein cholesterol (LDL-C)-lowering drugs for the prevention of cardiovascular disease [5]. Previous observational studies have shown that statins, ezetimibe, and PCSK9 inhibitors have protective effects against breast cancer and prostate cancer [6–8]. For example, Cauley et al.’s prospective cohort study found that women who were using statins or other lipid-lowering drugs had a 72% and 63% reduced risk of breast cancer compared with those who were not using, respectively [6]. In addition, a meta-analysis of 27 observational studies found that statin use significantly reduced the risk of prostate cancer [7]. However, there have been some studies reporting contradictory results to the above studies with no protective effects of them for the two cancers [9–11]. Both breast cancer and prostate cancer are multigenic, multifactorial and complex trait diseases, and there may be some factors confounding the study results in observational studies. Therefore, some confounding factors may lead to inconsistent results in some observational studies on associations of HMG-CoA reductase, NPC1L1 and PCSK9 with the risks of breast cancer and prostate cancer.

Mendelian randomization (MR) method may investigate the potential causal effect of an exposure of interest on the risk of disease by utilizing genetic variants as a proxy [12, 13]. Potential unmeasured confounders and reverse causation can be minimized in MR study due to random inheritance of parental genetic variants at conception [12, 13], while two-sample MR has better methodological advantages with two separate population studies [14]. Herein, we conducted a two-sample MR study to assess the causal associations of several LDL-C-lowering drug targets (HMG-CoA reductase, NPC1L1, and PCSK9) with breast cancer and prostate cancer.

## Methods

### Study design

**Fig. 1:**
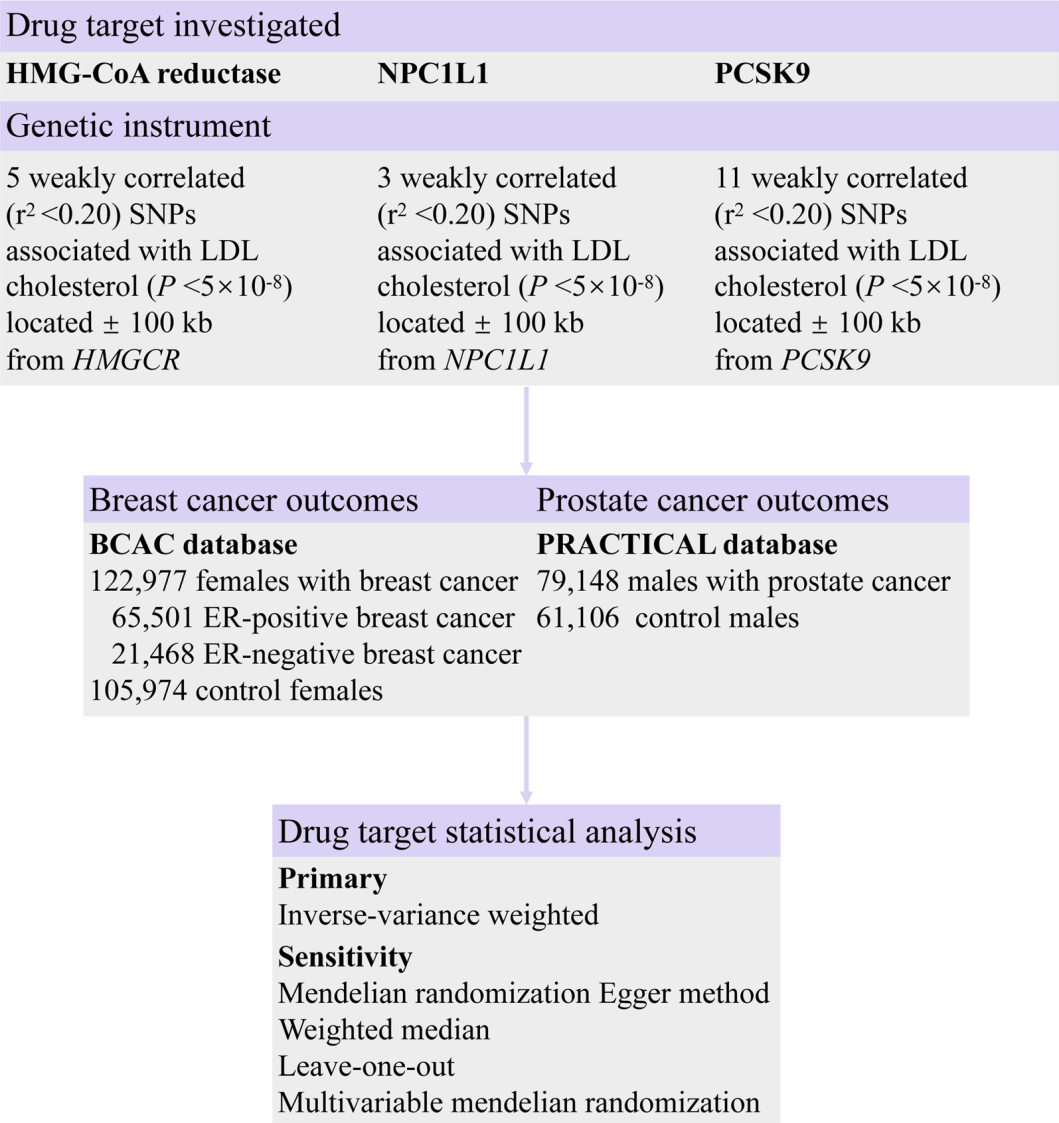
Genetic instrument construction, data sources, and analysis plan in a study of associations about the inhibition of HMG-CoA reductase, NPC1L1 and PCSK9 on breast cancer and prostate cancer. HMG-CoA reductase, 3-hydroxy-3-methylglutaryl coenzyme A; NPC1L1, Niemann-Pick C1-Like 1; PCSK9, proprotein convertase subtilisin/kexin type 9; BCAC database, The Breast Cancer Association Consortium; PRACTICAL database, Prostate Cancer Association Group to Investigate Cancer-Associated Alterations in the Genome Consortium; ER, estrogen receptor

As illustrated in Fig. 1, we designed an MR study to systematically investigate associations between inhibition of HMG-CoA reductase, NPC1L1, and PCSK9 and the risks of breast cancer and prostate cancer. Summary-level data of SNPs as genetic instruments for HMG-CoA reductase, NPC1L1, and PCSK9 were obtained from previously large-scale GWASs of European ancestry [15]. The protocol and data collection were approved by the ethics committee of the original GWASs, and written informed consent was obtained from each participant before data collection.

### Genetic instruments of LDL-C-lowering drug targets

To generate genetic instruments to proxy HMG-CoA reductase, NPC1L1, and PCSK9, summary genetic association data were obtained from a GWAS meta-analysis of LDL-C levels in the Global Lipids Genetics Consortium (GLGC) [15]. The GLGC (up to 188,577 European individuals) included 93,982 from 37 studies genotyped with the Metabochip array and 94,595 from 23 GWAS cohorts excluding overlap with Metabochip cohorts [15]. In most of the included studies, blood lipid concentrations were measured in fasting blood specimens (after at least 8 h of fasting), and individuals undergoing lipid-lowering treatment were excluded when possible.

To proxy HMG-CoA reductase, 5 SNPs associated with LDL-C at the genome-wide significance level (*P* < 5.0 × 10^−8^) and within ± 100 kb windows of the gene region encoding *HMG-CoA* reductase were obtained. Similarly, 3 LDL-C related SNPs within ± 100 kb from *NPC1L1* and 11 LDL-C related SNPs within ± 100 kb from *PCSK9* were used to proxy NPC1L1 and PCSK9, respectively. To maximize instrument strength, SNPs used as proxies for each drug target were permitted to be in low linkage disequilibrium (*r*^*2*^ < 0.20) with each other to increase the proportion of variance in each respective drug target explained by the instrument. The characteristics of genetic variants genetically proxied HMG-CoA reductase, NPC1L1 and PCSK9 are shown in Table 1.

**Table 1:** Characteristics of LDL-C-lowering genetic variants proxied for inhibition of HMG-CoA reductase, NPC1L1, and PCSK9

SNP^a^	Nearest gene	Position (build 37)	EA	NEA	EAF	Beta	Se	*P* value
*HMG-CoA reductase*								
rs12916	HMGCR	chr5:74656539	T	C	0.57	− 0.073	0.004	7.8e−78
rs10515198	HMGCR	chr5:74641560	G	A	0.90	− 0.060	0.006	6.0e−22
rs12173076	CERT1	chr5:74697050	T	G	0.88	− 0.065	0.006	2.3e−27
rs3857388	HMGCR	chr5:74620377	T	C	0.87	− 0.042	0.006	2.2e−11
rs7711235	ANKRD31	chr5:74540397	A	G	0.73	− 0.038	0.006	5.0e−10
*NPC1L1*								
rs2073547	NPC1L1	chr7:44582331	A	G	0.81	− 0.049	0.005	1.9e−21
rs217386	DDX56	chr7:44600695	A	G	0.41	− 0.036	0.004	1.2e−19
rs7791240	DDX56	chr7:44602589	T	C	0.91	− 0.043	0.007	1.8e−10
*PCSK9*								
rs11591147	PCSK9	chr1:55505647	T	G	0.02	− 0.497	0.018	8.6e−143
rs11206510	PCSK9	chr1:55496039	C	T	0.15	− 0.083	0.005	2.4e−53
rs2479409	PCSK9	chr1:55504650	A	G	0.67	− 0.064	0.004	2.5e−50
rs585131	PCSK9	chr1:55524116	C	T	0.18	− 0.064	0.005	2.7e−35
rs11206514	PCSK9	chr1:55516004	C	A	0.39	− 0.051	0.004	1.0e−32
rs2495477	PCSK9	chr1:55518467	G	A	0.40	− 0.064	0.005	7.3e−30
rs572512	PCSK9	chr1:55517344	C	T	0.65	− 0.048	0.005	5.3e−26
rs2479394	BSND	chr1:55486064	A	G	0.72	− 0.039	0.004	1.6e-19
rs12067569	PCSK9	chr1:55528629	G	A	0.97	− 0.089	0.010	2.0e−17
rs10493176	USP24	chr1:55538552	G	T	0.11	− 0.078	0.010	2.5e−14
rs11583974	USP24	chr1:55551718	G	A	0.97	− 0.065	0.012	4.0e−09

HMG-CoA reductase (HMGCR), 3-hydroxy-3-methylglutaryl coenzyme A; NPC1L1, Niemann-Pick C1-Like 1; PCSK9, proprotein convertase subtilisin/kexin type 9; SNP, Single-Nucleotide Polymorphism; EA, Effect Allele; NEA, Non-effect Allele; EAF, Effect Allele Frequency

^a^LDL-C associated SNPs (*P* < 5.0 × 10^–8^) in low linkage disequilibrium (*r*^2^ < 0.20) with each other and within ± 100 kb windows of gene region encoding HMG-CoA reductase, NPC1L1, and PCSK9 were used as genetic instruments (adapted from Willer et al. [15])

### Data sources for outcomes

Genetic association data of breast cancer, ER-positive breast cancer, and ER-negative breast cancer were obtained from a GWAS in the Breast Cancer Association Consortium (BCAC). The BCAC is an international collaboration initiated in 2005 to study genetic susceptibility to breast cancer, including 122,977 female cases and 105,974 female controls of European ancestry [16]. In BCAC, breast cancer cases (i.e., ER-positive breast cancer, ER-negative breast cancer, etc.) were mostly from the hospital and Cancer Registry [17]. The SNP-prostate cancer risk estimates were obtained from the Prostate Cancer Association Group to Investigate Cancer Associated Alterations in the Genome (PRACTICAL) consortium, which included genomic data of 79,148 European prostate cancer cases and 61,106 European controls [18]. Our analysis includes overall prostate cancer, which can be classified into several clinically relevant strata (e.g., T1, T2, T3, and M1) with the Gleason score and prostate specific antigen [18]. In the present study, the primary outcomes were breast cancer and prostate cancer, and the secondary outcomes included ER-positive breast cancer and ER-negative breast cancer.

### Statistical analysis

The strength of the genetic instruments of LDL-C-lowering drug targets was evaluated using the F-statistic [19], calculated by the equation *F* = (*N* − *K* − *1*) × *R*^2^/*K* × (1 − *R*^2^), where *R*^2^, calculated by using the package gtx in R (version 4.1.0; R Development Core Team), was the proportion of variation in HMG-CoA reductase, NPC1L1, or PCSK9 explained by the SNPs, N was the sample size, and K was the number of SNPs in genetically proxied inhibition of HMG-CoA reductase, NPC1L1, or PCSK9. Conventionally, an F statistic of at least 10 was indicative of evidence against weak instrument bias [19]. In addition, an online web tool named mRnd (https://shiny.cnsgenomics.com/mRnd/) was used to calculate the statistical power in this MR study [20].

In the main analysis, we used the inverse-variance weighted (IVW) MR method to estimate the effect of genetically proxied HMG-CoA reductase, NPC1L1, and PCSK9 on breast cancer and prostate cancer [21]. The heterogeneity between SNPs was evaluated by Cochran’s Q test [22]. If heterogeneity existed, random-effect IVW models were used and otherwise fixed-effect IVW models. To assess the robustness of the findings in the main analysis, we also performed a series of sensitivity analyses due to their resilience to violations of certain assumptions underlying the MR study. Firstly, we conducted MR-Egger regression analysis, of which the intercept term could be interpreted as pleiotropy across all variants [23, 24]. Secondly, we employed the weighted median approach to provide reliable estimates, in which analysis of the MR estimates was robust when < 50% of genetic variants were invalid [25]. Thirdly, iterative leave-one-out analysis was used to explore outlying or pleiotropic genetic variants by leaving each of them out of the MR analysis in turn [24, 26]. Finally, given the associations of LDL-C-lowering target instruments with body mass index and age at menarche [27], we further performed MR analysis with multivariable adjustment to estimate the relatively direct effects of LDL-C-lowering target on the risk of breast cancer (adjusting for body mass index and age at menarche) and prostate cancer (adjusting for body mass index) [28–31].

All MR estimates were presented as odds ratios (ORs) and were scaled up from individual SNP-level effects on LDL-C levels to reflect the equivalent of a 1-mmol/L (38.7-mg/dL) reduction in LDL-C levels. For the two primary outcomes (breast cancer and prostate cancer), all statistical tests were 2-sided, and a significance threshold was set at *P* < 0.008 (Bonferroni-correction significance threshold calculated as 0.05 divided by 6 [3 drug targets against 2 outcomes]). For the secondary outcome (ER-positive breast cancer and ER-negative breast cancer), an observed 2-sided *P* < 0.05 was considered to be statistically significant because these analyses were only exploratory analyses. All analyses were conducted with packages named TwoSampleMR, MendelianRandomization, and gtx in R software (version 4.1.0; R Development Core Team).

## Results

The strength of genetic instruments for LDL-C-lowering drug targets (HMG-CoA reductase, NPC1L1, and PCSK9) and the statistical power of this MR analysis are presented in Additional file 1: Table S1. The F statistics for the association of genetic instruments with HMG-CoA reductase, NPC1L1, and PCSK9 ranged from 71.63 to 195.81, suggesting that there is little instrument bias in the present MR study.

### Associations of LDL-C-lowering drug targets with breast cancer

**Fig. 2:**
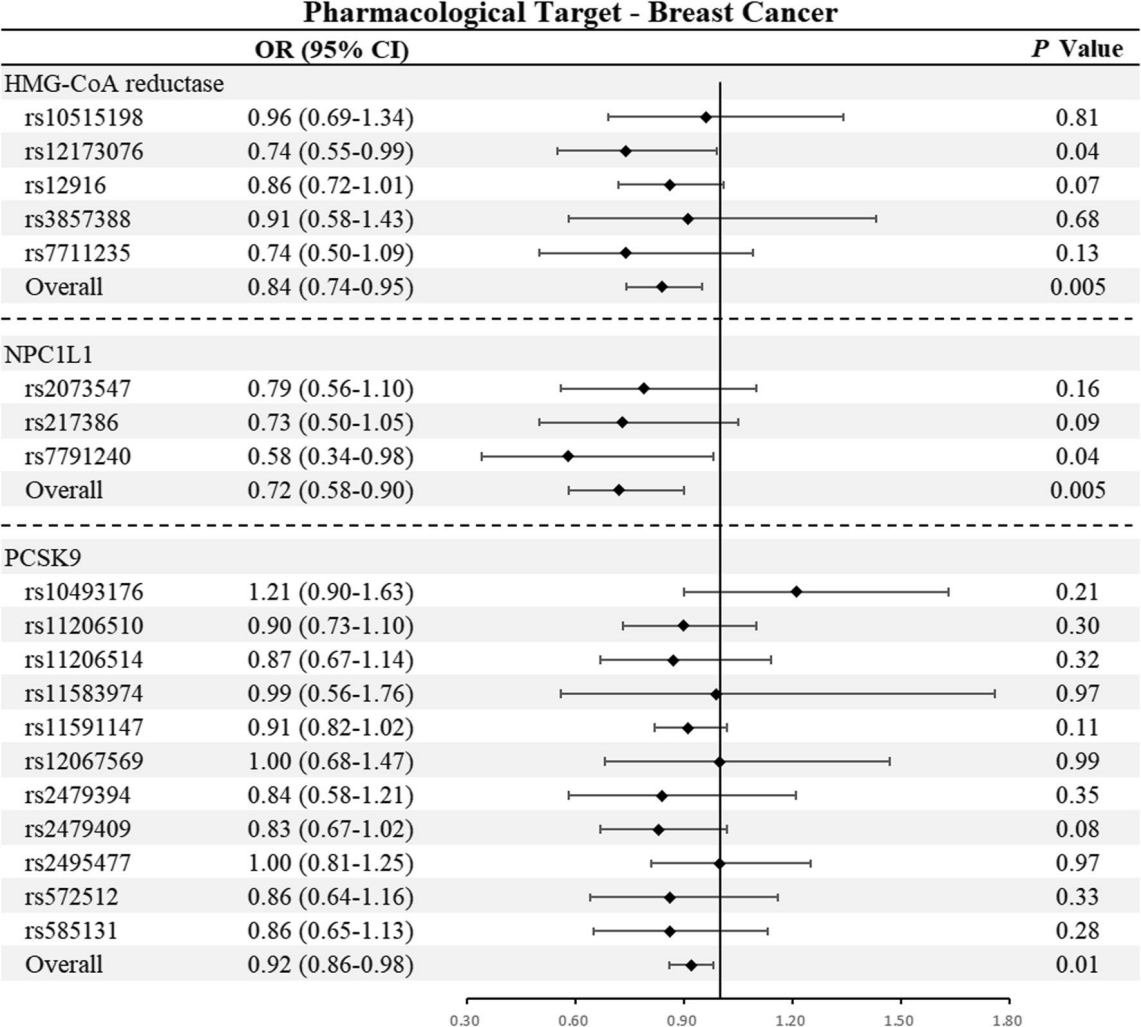
Mendelian randomization estimates of the associations between inhibition of HMG-CoA reductase, NPC1L1, and PCSK9 with breast cancer among women. HMG-CoA reductase, 3-hydroxy-3-methylglutaryl coenzyme A Reductase; NPC1L1, Niemann-Pick C1-Like 1; PCSK9, proprotein convertase subtilisin/kexin type 9; CI, confidence interval. An observed 2-sided *P* < 0.008 (Bonferroni-correction significance threshold calculated as 0.05 divided by 6 [3 drug targets against 2 outcomes]) was considered to be statistically significant

In light of Cochran’s Q test, the fixed-effect IVW models were used in the main analysis of breast cancer due to the lack of heterogeneity (Additional file 1: Table S2). As shown in Table 2 and Fig. 2, genetically proxied inhibition of HMG-CoA reductase (OR: 0.84; 95% CI 0.74–0.95; *P* = 0.005) and NPC1L1 (OR: 0.72; 95% CI 0.58–0.90; *P* = 0.005) equivalent to a 1-mmol/L (38.7 mg/dL) reduction in LDL-C was significantly associated with a reduced risk of breast cancer, respectively. However, the results showed no significant relationship between genetically determined inhibition of PCSK9 and the risk of breast cancer, although there was a trend of reduced risk of breast cancer (OR: 0.92; 95% CI 0.86–0.98; *P* = 0.01). In addition, further analysis showed that genetically proxied inhibition of HMG-CoA reductase and NPC1L1 were mainly associated with lower risk of ER-positive breast cancer (HMG-CoA reductase: OR: 0.82, *P* = 0.008; NPC1L1: OR: 0.66, *P* = 0.002) but not ER-negative breast cancer (Additional file 1: Table S3).

**Table 2:** Associations between genetically proxied inhibition of HMG-CoA reductase, NPC1L1, and PCSK9 and breast cancer and prostate cancer

Outcome	Case, No	OR (95% CI)	*P* value^a^
*HMG-CoA reductase*			
Breast cancer	122,977	0.84 (0.74–0.95)	0.005
Prostate cancer	79,148	0.85 (0.73–1.00)	0.05
*NPC1L1*			
Breast cancer	122,977	0.72 (0.58–0.90)	0.005
Prostate cancer	79,148	1.23 (0.92–1.63)	0.16
*PCSK9*			
Breast cancer	122,977	0.92 (0.86–0.98)	0.01
Prostate cancer	79,148	0.81 (0.73–0.90)	4.52e−05

HMG-CoA reductase, 3-hydroxy-3-methylglutaryl coenzyme A; NPC1L1, Niemann-Pick C1-Like 1; PCSK9, proprotein convertase subtilisin/kexin type 9; OR, Odds Ratio; CI, confidence interval

^a^Significance threshold was set at *P* < 0.008 (Bonferroni-correction significance threshold calculated as 0.05 divided by 6 [3 drug targets against 2 outcomes])

In the sensitivity analyses, MR-Egger showed no evidence of directional pleiotropy for the associations between inhibition of HMG-CoA reductase (odds [intercept]: 1.00; *P* = 0.77), NPC1L1 (odds [intercept]: 0.99; *P* = 0.84), and PCSK9 (odds [intercept]: 1.00; *P* = 0.72) and breast cancer (Additional file 1: Table S4). The results of leave-one-out sensitivity analyses showed that no individual SNP substantially drove the associations of HMG-CoA reductase and NPC1L1 with breast cancer (Additional file 1: Table S5).

### Associations of LDL-C-lowering drug targets with prostate cancer

**Fig. 3:**
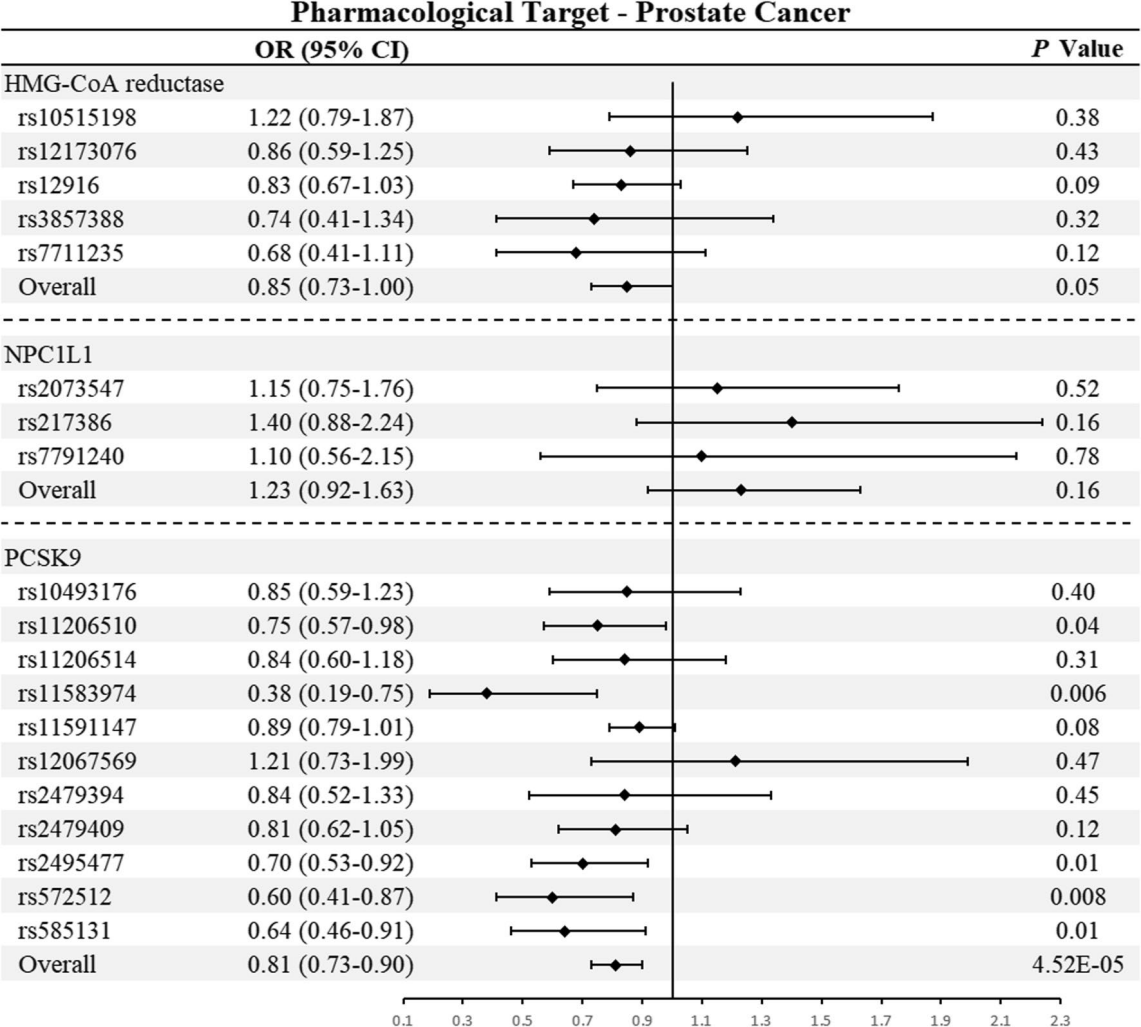
Mendelian randomization estimates of the associations between inhibition of HMG-CoA reductase, NPC1L1, and PCSK9 with prostate cancer among men. HMG-CoA reductase, 3-hydroxy-3-methylglutaryl coenzyme A Reductase; NPC1L1, Niemann-Pick C1-Like 1; PCSK9, proprotein convertase subtilisin/kexin type 9; CI, confidence interval. An observed 2-sided *P* < 0.008 (Bonferroni-correction significance threshold calculated as 0.05 divided by 6 [3 drug targets against 2 outcomes]) was considered to be statistically significant

In the main analysis of prostate cancer, fixed-effect IVW models were used for HMG-CoA reductase and NPC1L1 because of no heterogeneity, while random effect IVW model was also used for PCSK9 (Additional file 1: Table S2). As shown in Table 2 and Fig. 3, genetically proxied PCSK9 inhibition was associated with a reduced risk of prostate cancer (OR: 0.81; 95% CI 0.73–0.90; *P* = 4.52 × 10^–5^). In contrast, genetically proxied inhibition of HMG-CoA reductase (OR: 0.85; 95% CI 0.73–1.00; *P* = 0.05) or NPC1L1 (OR: 1.23; 95% CI 0.92–1.63; *P* = 0.16) was not significantly associated with prostate cancer.

In light of the MR-Egger regression findings (Table 2), we found no evidence against directional pleiotropy for the associations between genetically determined inhibition of HMG-CoA reductase (odds [intercept]: 1.00; *P* = 0.52), NPC1L1 (odds [intercept]: 1.03; *P* = 0.64), and PCSK9 (odds [intercept]: 0.99; *P* = 0.09) and the risk of prostate cancer (Additional file 1: Table S4). In the sensitivity analysis applying the weighted median method, the association of genetically proxied PCSK9 inhibition with prostate cancer (OR: 0.85; 95% CI 0.76–0.95; *P* = 0.005; Additional file 1: Table S6) remained significant. Leave-one-out sensitivity analysis showed that the association between PCSK9 inhibition and prostate cancer was not substantially driven by any individual SNP (Additional file 1: Table S7).

### Multivariable MR analyses

The results of multivariable MR analysis on the associations of LDL-C-lowering drug targets with breast cancer and prostate cancer are presented in Table S8. In terms of breast cancer, multivariable MR analyses adjusting for body mass index and age at menarche suggested that genetically proxied inhibition of HMG-CoA reductase was associated with a reduced risk of breast cancer (OR: 0.85; 95% CI 0.75–0.95; *P* = 0.007). For prostate cancer, significant associations were observed for genetically proxied PCSK9 inhibition and low odds of prostate cancer (OR: 0.81; 95% CI 0.71–0.92; *P* = 0.002).

## Discussion

In this large-scale MR analysis of 122,977 cases and 105,974 controls for breast cancer and 79,148 cases and 61,106 controls for prostate cancer, we investigated the effect of three common LDL-C-lowering drug targets (HMG-CoA reductase, NPC1L1, and PCSK9) on the risks of breast cancer and prostate cancer. We found that genetically proxied inhibition of HMG-CoA reductase and NPC1L1 but not PCSK9 were significantly associated with reduced risk of breast cancer (mainly ER-positive breast cancer), while only genetically determined PCSK9 inhibition was associated with low odds of prostate cancer. These findings suggested that there were possible mechanism-specific protective effects of statins (targeting HMG-CoA reductase) and ezetimibe (targeting NPC1L1) on breast cancer, whereas PCSK9 inhibitors might have protective effect on prostate cancer.

In recent decades, observational studies investigating the effects of statins and alternate LDL-C-lowering drugs (ezetimibe and PCSK9 inhibitors) on the risks of breast cancer and prostate cancer have yielded inconsistent results [6, 7, 9, 10, 32]. In an analysis based on a multicenter cohort study involving 7,528 Caucasian females in the United States, the participants who used lipid-lowering drugs had a significantly reduced risk of developing breast cancer compared with those who did not use [6]. A meta-analysis of 27 observational studies showed that statins use significantly reduced the risk of prostate cancer by 7% [7]. The above two findings seemed to reveal the potential application possibility of lipid-lowering drugs in the prevention of breast cancer and prostate cancer. However, in an analysis based on the Cancer Prevention Study II Nutrition Cohort with a 10-year follow-up period, long-term use of cholesterol-lowering drugs were not associated with the risk of breast cancer and prostate cancer [10]. Lipid-lowering drugs have been widely used to prevent hyperlipidemia, atherosclerosis and cardiovascular disease in population. Clarifying the relationship between lipid-lowering drugs and breast cancer and prostate cancer has not only scientific significance, but also practical implications for the prevention of breast cancer and prostate cancer. The inconsistent results of the above studies may be due to the reason why observational study and conventional analysis are incapable of completely overcoming the confounding because of the polygenic and multifactorial characteristics of breast cancer and prostate cancer with complex traits.

With regard to the inherent bias of residual confounding and reverse causation in observational studies, MR is an increasingly used method to draw definitive conclusions regarding the causality of the association between exposures and diseases via considering genetic variants as instrumental variables [12–14]. MR leverages the random allocation of exposure-influencing genetic alleles and can avoid reverse-causality bias and minimize confounding by other determinants in a similar pattern as randomized controlled trials [33]. Recently, genetically proxied inhibition of HMG-CoA reductase but not NPC1L1 and PCSK9 was associated with lower odds of epithelial ovarian cancer in MR study, while no such MR study was currently available for breast cancer and prostate cancer [27]. In the present study, we first found that genetically proxied inhibition of HMG-CoA reductase and NPC1L1 were significantly associated with lower odds of breast cancer (especially ER-positive breast cancer), while the inhibition of PCSK9 was associated with reduced risk of prostate cancer.

There exists numerous important public health significances and clinical implications. From our findings, the problems that lipid lowering drugs maybe prevent breast cancer and prostate cancer in population deserve to be further studied, and even statins and ezetimibe may be recommended for the prevention of breast cancer in the population, especially ER-positive breast cancer, among females with hyperlipidemia. In contrast, PCSK9 inhibitors may be the preferred LDL-C-lowering drug among males with hyperlipidemia to prevent prostate cancer. Some randomized controlled trials for preventing cardiovascular disease have suggested that statins have provocative and unexpected benefits for reducing colorectal cancer and melanoma [34–36]. However, there have not been randomized controlled trials designed to study the effects of statins and ezetimibe on breast cancer and PCSK9 inhibitors on prostate cancer. Therefore, further well-designed randomized trials are warranted to verify the protective effects of statins and ezetimibe on breast cancer and PCSK9 inhibitors on prostate cancer. The validated findings will promote precise prevention and develop personalized treatment strategies for breast cancer and prostate cancer.

Several biological mechanisms may underlie the beneficial effect of LDL-C-lowering drugs on breast cancer and prostate cancer. HMG-CoA reductase inhibition may reduce intratumoural autocrine hormone production by lowering intracellular cholesterol, promote apoptosis breast cancer cells through inducing nitric oxide synthase expression [37, 38]. As the target of ezetimibe, inhibition of NPC1L1 can decrease the level of bile-derived cholesterol to inhibit the angiogenesis of breast tumors [39]. PCSK9 inhibition appeared to be implicated in prostate cancer by regulating the expression of squalene monooxygenase and lectin-like oxidized low-density lipoprotein receptor-1 [40–42]. In addition, the influence of hormones, sex differences in immunity, and complexity of the regulatory mechanism in tumor angiogenesis may also contribute to the different roles of stains, ezetimibe and PCSk9 inhibition in breast cancer and prostate cancer [39, 40, 43]. Further functional studies are warranted to investigate the mechanisms underlying the specific protective effects of certain lipid-lowering drugs on the breast cancer and prostate cancer.

This study had a number of important strengths. First, to the best of our knowledge, this is the first MR study to detect the causal effects of lipid-lowering drugs on breast cancer, breast cancer subtypes, and prostate cancer. Second, the present study used genetic variants within genes that encoded drug targets to proxy the potential effect of commonly prescribed LDL-C-lowering therapies, which could reflect the impact of life-long exposure on breast cancer or prostate cancer and avoid the drawback of limited exposure time and follow-up time in clinical trials or observational studies. Third, the summary statistics of lipid-lowering drugs, breast cancer and prostate cancer were collected from well-designed GWAS with large sample size, which enabled us to make causal inferences with high statistical power. Fourth, our study harnessed the homogeneity of European populations with respect to genetic background and external sociocultural determinants, decreasing spurious associations due to population stratification and other confounding factors [44]. Certainly, further studies included multi-ethnicity samples should be conducted to confirm our findings.

Our study also had several limitations. First, MR estimates represented the long-term modulation of drug targets on disease risk, which might suggest larger risk reductions per unit change in drug target compared with those obtained from drug administration over a relatively shorter duration. Therefore, if lipid-lowering drug treatment could lower the risk of breast cancer or prostate cancer, the magnitude of risk lowering achieved through taking lipid-lowering drugs might not correspond to the effect size observed in this MR study. Second, although we attempted to control for pleiotropy in the MR study, pleiotropy still represented a major challenge to decipher the roles of specific lipid-based pathways. In the present study, MR-Egger regression analysis showed no pleiotropic effects, indicating that the possibility of directional pleiotropy bias may be minimal. Third, MR analysis might be biased by potential violations of standard instrumental variable assumptions [23]. However, several sensitivity analyses in the present study observed no evidence of violations and further confirmed the robustness of the results in the main analysis.

## Conclusions

Genetically proxied inhibition of HMG-CoA reductase and NPC1L1 was significantly associated with lower odds of breast cancer (especially ER-positive breast cancer), while genetically proxied inhibition of PCSK9 was associated with reduced risk of prostate cancer. Further randomized controlled trials are needed to confirm the respective roles of these LDL-C-lowering drugs in breast cancer and prostate cancer.

## Supplementary Information

**Additional file 1**. Supplementary Online Content.

## Data Availability

Single-nucleotide polymorphisms (SNPs) in HMGCR, NPC1L1, and PCSK9 associated with LDL-C in a genome-wide association study (GWAS) meta-analysis from Global Lipids Genetics Consortium (GLGC; up to 188,577 European individuals) were used to proxy therapeutic inhibition of HMG-CoA reductase, NPC1L1, and PCSK9, respectively. Summary statistics for these SNPs were obtained from a GWAS meta-analysis of the Breast Cancer Association Consortium (BCAC; 228,951 European females) and from a Prostate Cancer Association Group to Investigate Cancer Associated Alterations in the Genome (PRACTICAL; 140,254 European males) consortium.

## Abbreviations

LDL-C: Low-density lipoprotein cholesterol
HMG-CoA: 3-Hydroxy-3-methylglutaryl coenzyme A
NPC1L1: Niemann-Pick C1-Like 1
PCSK9: Proprotein convertase subtilisin/kexin type 9
MR: Mendelian randomization
SNPs: Single-nucleotide polymorphisms
GWAS: Genome-wide association study
GLGC: Global Lipids Genetics Consortiumthe
BCAC: Breast Cancer Association Consortium
PRACTICAL: Prostate Cancer Association Group to Investigate Cancer Associated Alterations in the Genome consortium
ER: Estrogen receptor
IVW: Inverse-variance weighted
