# Supplementary material for: Associations of genetically proxied inhibition of HMG-CoA reductase, NPC1L1, and PCSK9 with breast cancer and prostate cancer
Source: Breast Cancer Res. 2022 Feb 12;24:12. doi: 10.1186/s13058-022-01508-0 (PMC8840684; doi:10.1186/s13058-022-01508-0)
Supplement: Supplementary file 1 — Additional file 1. Supplementary Online Content. [file 13058_2022_1508_MOESM1_ESM.docx]

**Supplementary Online Content**

**Table S1.** F-statistic estimates for genetic instruments and statistic power (%) estimates of lowing-lipid target with breast cancer, breast cancer subtypes, and prostate cancer

**Table S2.** Results of heterogeneity test and inverse-variance-weighted model used in the analysis

**Table S3.** Associations between genetically proxied inhibition of HMG-CoA reductase, NPC1L1, and PCSK9 and breast cancer subtypes in mendelian randomization statistical analyses

**Table S4.** Associations between genetically proxied inhibition of HMG-CoA reductase, NPC1L1, and PCSK9 and risk of breast cancer and prostate cancer in mendelian randomization Egger method

**Table S5.** Associations between genetically proxied inhibition of HMG-CoA reductase, NPC1L1, and PCSK9 and risk of breast cancer in the leave-one-out analysis

**Table S6.** Associations between genetically proxied inhibition of HMG-CoA reductase, NPC1L1, and PCSK9 and risk of breast cancer and prostate cancer in weighted median

**Table S7.** Associations between genetically proxied inhibition of HMG-CoA reductase, NPC1L1 and PCSK9 and risk of prostate cancer in the leave-one-out analysis

**Table S8.** Associations between genetically proxied inhibition of HMG-CoA reductase, NPC1L1, and PCSK9 and breast cancer and prostate cancer in multivariable mendelian randomization

**Table S1. F-statistic estimates for genetic instruments and statistic power (%) estimates of lowing-lipid target with breast cancer, breast cancer subtypes, and prostate cancer**

| **Outcome** | **HMG - CoA reductase^a^** | **NPC1L1^a^** | **PCSK9^a^** |
| --- | --- | --- | --- |
|  | **(F^b^=128.14)** | **(F^b^=71.63)** | **(F^b^=195.81)** |
| Breast cancer | 100 | 100 | 100 |
| ER-positive breast cancer | 100 | 100 | 100 |
| ER-negative breast cancer | 99 | 75 | 100 |
| Prostate cancer | 100 | 100 | 100 |

Abbreviations: HMG-CoA reductase, 3-hydroxy-3-methylglutaryl coenzyme A Reductase; NPC1L1, Niemann-Pick C1-Like 1; PCSK9, proprotein convertase subtilisin/kexin type 9, ER, estrogen receptor.

^a^ Power calculations represent statistical power to detect an odds ratio of OR=0.50 per 1mmol/L (38.7mg/dL) reduction in LDL cholesterol at a 5% false positive rate.

^b^ F-statistics that quantify the strength of the selected instrumental variables were done with the formula of F=(N-K-1)×R^2^/K×(1-R^2^), where R^2^ is the proportion of variation in HMG-CoA reductase, NPC1L1, or PCSK9 explained by the SNPs, N is the sample size, and K is the number of SNPs in genetically proxied inhibition of HMG-CoA reductase, NPC1L1, or PCSK9.

**Table S2. Results of heterogeneity test and inverse-variance-weighted model used in the analysis**

| **Item** | **Q value** | **Model** |
| --- | --- | --- |
| HMG-CoA Reductase | | |
| Breast cancer | 1.90 | fixed effect model |
| ER-positive breast cancer | 3.65 | fixed effect model |
| ER-negative breast cancer | 2.41 | fixed effect model |
| Prostate cancer | 3.70 | fixed effect model |
| NPC1L1 | | |
| Breast cancer | 0.95 | fixed effect model |
| ER-positive breast cancer | 1.12 | fixed effect model |
| ER-negative breast cancer | 0.32 | fixed effect model |
| Prostate cancer | 0.49 | fixed effect model |
| PCSK9 | | |
| Breast cancer | 5.93 | fixed effect model |
| ER-positive breast cancer | 4.56 | fixed effect model |
| ER-negative breast cancer | 13.06 | fixed effect model |
| Prostate cancer | 15.14 | random effect model |

Abbreviations: HMG-CoA reductase, 3-hydroxy-3-methylglutaryl coenzyme A Reductase; NPC1L1, Niemann-Pick C1-Like 1; PCSK9, proprotein convertase subtilisin/kexin type 9, ER, estrogen receptor.

**Table S3. Associations between genetically proxied inhibition of HMG-CoA reductase, NPC1L1, and PCSK9 and breast cancer subtypes in mendelian randomization statistical analyses**

| **Target** | **Parameter** | **HMG-CoA reductase** | | **NPC1L1** | | | **PCSK9** | |
| --- | --- | --- | --- | --- | --- | --- | --- | --- |
|  |  | **OR (95% CI)** | ***P* value^b^** | **OR (95% CI)** | | ***P* value^b^** | **OR (95% CI)** | ***P* value^b^** |
| ER-positive breast cancer (*n*=69 501/10 5974) | | | | | | | | |
| IVW | OR | 0.82 (0.71-0.95) | 0.008 | 0.66 (0.50-0.86) | 0.002 | | 0.94 (0.86-1.01) | 0.11 |
| MR Egger | OR | 0.96 (0.52-1.78) | 0.91 | 0.82 (0.11-6.05) | 0.88 | | 0.98 (0.85-1.12) | 0.76 |
|  | Odds (intercept) | 0.99 (0.96-1.03) | 0.64 | 0.99 (0.91-1.01) | 0.86 | | 1.00 (0.99-1.01) | 0.46 |
| Weighted median | OR | 0.80 (0.68-0.94) | 0.008 | 0.69 (0.51-0.94) | 0.02 | | 0.94 (0.85-1.05) | 0.30 |
| MVMR^a^ | OR | 0.83 (0.69-1.00) | 0.05 | 0.62 (0.42-0.92) | 0.02 | | 0.94 (0.86-1.02) | 0.11 |
| ER-negative breast cancer (*n*=21 468/105 974) | | | | | | | | |
| IVW | OR | 0.85 (0.68-1.06) | 0.14 | 0.87 (0.58-1.31) | 0.51 | | 0.91(0.80-1.03) | 0.14 |
| MR Egger | OR | 0.80 (0.33-1.95) | 0.66 | 1.34 (0.07-24.79) | 0.88 | | 0.90 (0.69-1.17) | 0.45 |
|  | Odds (intercept) | 1.00 (0.95-1.06) | 0.91 | 0.98 (0.87-1.11) | 0.82 | | 1.00 (0.98-1.02) | 0.90 |
| Weighted median | OR | 0.85 (0.65-1.12) | 0.25 | 0.88 (0.56-1.40) | 0.60 | | 0.88 (0.75-1.05) | 0.16 |
| MVMR^a^ | OR | 0.86 (0.69-1.06) | 0.16 | 0.84 (0.57-1.25) | 0.40 | | 0.91 (0.80-1.03) | 0.12 |

Abbreviations: ER, estrogen receptor; HMG-CoA reductase, 3-hydroxy-3-methylglutaryl coenzyme A Reductase; NPC1L1, Niemann-Pick C1-Like 1; PCSK9, proprotein convertase subtilisin/kexin type 9; OR, Odds ratios; CI, confidence interval; IVW, inverse-variance weighted; MVMR, multivariable mendelian randomization.

^a^ Adjusted for body mass index, age at menarche.

^b^An observed 2-sided *P* <0.05 was considered to be statistically significant because these analyses were only exploratory analyses.

**Table S4. Associations between genetically proxied inhibition of HMG-CoA reductase, NPC1L1, and PCSK9 and risk of breast cancer and prostate cancer in mendelian randomization Egger method**

| **Target** | **Parameter** | **OR** | **95% CI** | ***P* value^a^** |
| --- | --- | --- | --- | --- |
| HMG-CoA reductase |  |  |  |  |
| breast cancer | OR | 0.91 | 0.56-1.48 | 0.72 |
|  | Odds(intercept) | 1.00 | 0.97-1.02 | 0.77 |
| prostate cancer | OR | 1.08 | 0.57-2.06 | 0.83 |
|  | Odds(intercept) | 1.00 | 0.95-1.02 | 0.52 |
| NPC1L1 | | | |  |
| breast cancer | OR | 0.90 | 0.18-4.52 | 0.92 |
|  | Odds(intercept) | 0.99 | 0.93-1.06 | 0.84 |
| prostate cancer | OR | 0.64 | 0.08-4.94 | 0.74 |
|  | Odds(intercept) | 1.03 | 0.94-1.12 | 0.64 |
| PCSK9 | | | |  |
| breast cancer | OR | 0.93 | 0.83-1.05 | 0.28 |
|  | Odds(intercept) | 1.00 | 0.99-1.01 | 0.72 |
| prostate cancer | OR | 0.91 | 0.78-1.06 | 0.26 |
|  | Odds(intercept) | 0.99 | 0.98-1.00 | 0.09 |

Abbreviations: HMG-CoA reductase, 3-hydroxy-3-methylglutaryl coenzyme A Reductase; NPC1L1, Niemann-Pick C1-Like 1; PCSK9, proprotein convertase subtilisin/kexin type 9; SNP， Single-Nucleotide Polymorphism; OR, Odds ratios; CI, confidence interval.

^a^A 2-sided *P*<0.05 was considered as suggestive evidence for potential directional pleiotropy in the MR-Egger regression method.

**Table S5. Associations between genetically proxied inhibition of HMG-CoA reductase, NPC1L1, and PCSK9 and risk of breast cancer in the leave-one-out analysis**

| **Target** | **SNP removed** | **OR** | **95% CI** | ***P* value^a^** |
| --- | --- | --- | --- | --- |
| HMG-CoA reductase |  |  |  |  |
|  | rs10515198 | 0.82 | 0.72-0.94 | 0.003 |
|  | rs12173076 | 0.86 | 0.75-0.99 | 0.03 |
|  | rs12916 | 0.82 | 0.69-0.98 | 0.03 |
|  | rs3857388 | 0.83 | 0.73-0.95 | 0.005 |
|  | rs7711235 | 0.85 | 0.75-0.97 | 0.01 |
|  | ALL | 0.84 | 0.74-0.95 | 0.005 |
| NPC1L1 |  |  |  |  |
|  | rs2073547 | 0.67 | 0.50-0.91 | 0.01 |
|  | rs217386 | 0.72 | 0.54-0.95 | 0.02 |
|  | rs7791240 | 0.76 | 0.59-0.97 | 0.03 |
|  | ALL | 0.72 | 0.58-0.90 | 0.005 |
| PCSK9 |  |  |  |  |
|  | rs10493176 | 0.90 | 0.84-0.97 | 0.004 |
|  | rs11206510 | 0.92 | 0.86-0.99 | 0.02 |
|  | rs11206514 | 0.92 | 0.86-0.99 | 0.02 |
|  | rs11583974 | 0.92 | 0.86-0.98 | 0.01 |
|  | rs11591147 | 0.92 | 0.84-1.00 | 0.05 |
|  | rs12067569 | 0.92 | 0.85-0.98 | 0.01 |
|  | rs2479394 | 0.92 | 0.86-0.98 | 0.02 |
|  | rs2479409 | 0.93 | 0.86-1.00 | 0.04 |
|  | rs2495477 | 0.91 | 0.85-0.97 | 0.008 |
|  | rs572512 | 0.92 | 0.86-0.99 | 0.02 |
|  | rs585131 | 0.92 | 0.86-0.99 | 0.02 |
|  | ALL | 0.92 | 0.86-0.98 | 0.01 |

Abbreviations: HMG-CoA reductase, 3-hydroxy-3-methylglutaryl coenzyme A Reductase; NPC1L1, Niemann-Pick C1-Like 1; PCSK9, proprotein convertase subtilisin/kexin type 9; SNP， Single-Nucleotide Polymorphism; OR, Odds ratios; CI, confidence interval.

^a^ Significance threshold was set at *P*<0.008 (Bonferroni-correction significance threshold calculated as 0.05 divided by 6 [3 drug targets against 2 outcomes]).

**Table S6. Associations between genetically proxied inhibition of HMG-CoA reductase, NPC1L1, and PCSK9 and risk of breast cancer and prostate cancer in weighted median**

| **Target** | **OR** | **95% CI** | ***P* value^a^** |
| --- | --- | --- | --- |
| HMG-CoA reductase |  |  |  |
| breast cancer | 0.85 | 0.73-0.98 | 0.03 |
| prostate cancer | 0.84 | 0.70-1.00 | 0.05 |
| NPC1L1 |  |  |  |
| breast cancer | 0.75 | 0.58-0.97 | 0.03 |
| prostate cancer | 1.20 | 0.88-1.64 | 0.25 |
| PCSK9 |  |  |  |
| breast cancer | 0.91 | 0.83-0.99 | 0.04 |
| prostate cancer | 0.85 | 0.76-0.95 | 0.005 |

Abbreviations: HMG-CoA reductase, 3-hydroxy-3-methylglutaryl coenzyme A Reductase; NPC1L1, Niemann-Pick C1-Like 1; PCSK9, proprotein convertase subtilisin/kexin type 9; OR, Odds ratios; CI, confidence interval.

^a^ Significance threshold was set at *P*<0.008 (Bonferroni-correction significance threshold calculated as 0.05 divided by 6 [3 drug targets against 2 outcomes]).

**Table S7. Associations between genetically proxied inhibition of HMG-CoA reductase, NPC1L1 and PCSK9 and risk of prostate cancer in the leave-one-out analysis**

| **Target** | **SNP removed** | **OR** | **95% CI** | ***P* value^a^** |
| --- | --- | --- | --- | --- |
| HMG-CoA reductase |  |  |  |  |
|  | rs10515198 | 0.81 | 0.68-0.96 | 0.01 |
|  | rs12173076 | 0.85 | 0.70-1.03 | 0.10 |
|  | rs12916 | 0.88 | 0.69-1.13 | 0.32 |
|  | rs3857388 | 0.86 | 0.72-1.03 | 0.10 |
|  | rs7711235 | 0.88 | 0.74-1.03 | 0.12 |
|  | ALL | 0.85 | 0.73-1.00 | 0.05 |
| NPC1L1 |  |  |  |  |
|  | rs2073547 | 1.29 | 0.88-1.90 | 0.19 |
|  | rs217386 | 1.14 | 0.79-1.62 | 0.49 |
|  | rs7791240 | 1.26 | 0.92-1.72 | 0.16 |
|  | ALL | 1.23 | 0.92-1.63 | 0.16 |
| PCSK9 |  |  |  |  |
|  | rs10493176 | 0.81 | 0.72-0.90 | 1.27e-04 |
|  | rs11206510 | 0.82 | 0.73-0.91 | 3.44e-04 |
|  | rs11206514 | 0.81 | 0.72-0.90 | 1.46e-04 |
|  | rs11583974 | 0.82 | 0.75-0.89 | 1.12e-05 |
|  | rs11591147 | 0.75 | 0.67-0.85 | 3.63e-06 |
|  | rs12067569 | 0.80 | 0.72-0.88 | 1.10e-05 |
|  | rs2479394 | 0.81 | 0.72-0.90 | 1.28e-04 |
|  | rs2479409 | 0.81 | 0.72-0.91 | 2.38e-04 |
|  | rs2495477 | 0.82 | 0.74-0.91 | 3.41e-04 |
|  | rs572512 | 0.82 | 0.74-0.91 | 1.18e-04 |
|  | rs585131 | 0.82 | 0.74-0.91 | 1.87e-04 |
|  | ALL | 0.81 | 0.73-0.90 | 4.52e-05 |

Abbreviations: HMG-CoA reductase, 3-hydroxy-3-methylglutaryl coenzyme A Reductase; NPC1L1, Niemann-Pick C1-Like 1; PCSK9, proprotein convertase subtilisin/kexin type 9; SNP， Single-Nucleotide Polymorphism; OR, Odds ratios; CI, confidence interval.

^a^ Significance threshold was set at *P*<0.008 (Bonferroni-correction significance threshold calculated as 0.05 divided by 6 [3 drug targets against 2 outcomes]).

**Table S8. Associations between genetically proxied inhibition of HMG-CoA reductase, NPC1L1, and PCSK9 and breast cancer and prostate cancer in multivariable mendelian randomization**

| **Target** | **OR** | **95% CI** | ***P* value^c^** |
| --- | --- | --- | --- |
| HMG-CoA reductase |  |  |  |
| breast cancer^a^ | 0.85 | 0.75-0.95 | 0.007 |
| prostate cancer^b^ | 0.83 | 0.71-0.96 | 0.01 |
| NPC1L1 |  |  |  |
| breast cancer^a^ | 0.69 | 0.50-0.94 | 0.02 |
| prostate cancer^b^ | 1.25 | 0.94-1.66 | 0.12 |
| PCSK9 |  |  |  |
| breast cancer^a^ | 0.92 | 0.86-0.98 | 0.01 |
| prostate cancer^b^ | 0.81 | 0.71-0.92 | 0.002 |

Abbreviations: HMG-CoA reductase, 3-hydroxy-3-methylglutaryl coenzyme A Reductase; NPC1L1, Niemann-Pick C1-Like 1; PCSK9, proprotein convertase subtilisin/kexin type 9; OR, Odds ratios; CI, confidence interval.

^a^ Adjusted for body mass index, age at menarche.

^b^ Adjusted for body mass index.

^c^ Significance threshold was set at *P*<0.008 (Bonferroni-correction significance threshold calculated as 0.05 divided by 6 [3 drug targets against 2 outcomes]).
